# Crohn’s Disease Previously Mistreated as Intestinal Tuberculosis Complicated with Ileocecal Lump: A Case Report

**DOI:** 10.31729/jnma.8534

**Published:** 2024-04-30

**Authors:** Shriya Sharma, Pasang Sherpa, Ganesh Giri

**Affiliations:** 1 Division of Advanced Heart Failure and Transplant, Mayo Clinic, Jacksonville, Florida, USA; 2 Division of Quantitative Health Sciences, Mayo Clinic, Jacksonville, Florida, USA

**Keywords:** *colectomy*, *crohn's disease*, *tuberculosis*

## Abstract

In Southeast Asia, the higher prevalence of Intestinal tuberculosis (TB) challenges the diagnosis of Crohn's disease (CD) due to their overlapping symptoms. This case involves a 25-year-old male misdiagnosed with Intestinal tuberculosis presenting with abdominal pain, weight loss, and bowel ulceration. Recurrence after anti-tubercular therapy led to further investigation paving to right hemicolectomy and histopathological analysis confirming Crohn's disease. This case highlights the complexity of the diagnosis of Crohn's disease in tuberculosis-prevalent areas, stressing the clinical importance, advanced diagnostics tools, and multidisciplinary approach for effective intervention.

## INTRODUCTION

Crohn's Disease (CD) and intestinal tuberculosis (TB) are chronic granulomatous inflammatory disorders of the gastrointestinal tract and there is increasing incidence of CD in the world.^1,2^ Both present with abdominal pain, diarrhea, weight loss, blood/mucus in stool with complications of intestinal fistula, perforation, obstruction, abscesses, etc.^3,4^ It is difficult to distinguish between them due to their comparable endoscopic, radiological, and clinical presentations.^5^ Thus, patients with clinical symptoms and ulceration in bowel are often diagnosed with intestinal tuberculosis.^6^ We present a case in line with SCARE (Surgical Case Report) 2020 guideline in which a patient was misdiagnosed with intestinal tuberculosis, complicated by ileocecal lump followed by right hemicolectomy.^7^

## CASE REPORT

A 25-year-old man with no significant past medical history came to the outpatient department with complaints of low-grade fever for one month and unintentional weight loss of approximately 8 kilograms in the last 1 month along with pain in the right lower quadrant for 20 days. He felt feverish most of the time but the temperature was always below 100 degrees Fahrenheit. The pain was on the right lower quadrant, dull aching, and continuous, which aggravated around 90 minutes after his meal. The patient also experienced altered bowel habits as he had to evacuate his bowel after every meal. Occasionally, stool was mixed with the blood without mucus and defecation was associated with tenesmus. For the above-mentioned complaints, he initially visited the nearby pharmacy and took some medications after which he felt some relief for a few days but his symptoms reoccurred, so he decided to visit the hospital.

On examination, his abdomen was soft and nontender with no palpable masses or organomegaly. The perianal examination was also normal. Blood chemistry results showed haemoglobin level 12.7 (Reference: 13-18 gm/dl), erythrocyte segmentation ratio (ESR) 25 (Reference: 0-10 mm/first hour), prothrombin time 17 (Reference: 11-15 seconds), platelets count was increased to 465,000 (Reference: 150,000-450,000/ cumm), mean corpuscular haemoglobin was 26 (Reference: 27-33 pg) and mean corpuscular volume (MCV) was 77 (Reference: 80-100 fl).

**Figure 1.**
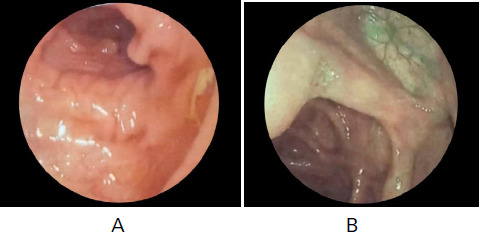
A- Multiple small ulcers with surrounding edema in the caecum and B- Few ulcers involving the ileocecal valve region.

A colonoscopy was done which showed multiple small ulcers with surrounding edema in the caecum and a few ulcers involving the ileocecal valve region (Figure 1). Biopsy taken from the caecal region showed ulceration with exudate, patchy colitis with mild crypt distortion, neural and ganglion cell hyperplasia, scattered giant cells, and focal epithelioid small granulomas with the lymphoid aggregate. Although the Acid-Fast Bacilli stain was negative, tuberculosis or other infectious colitis couldn't be completely excluded. Computed Tomography (CT) scan of the abdomen showed circumferential wall thickening of approximately 55 mm long segment of distal ileum with soft tissue stranding of adjacent mesentery with hypertrophied mesenteric vessels and multiple enlarged mesenteric lymph nodes. Based on the history and laboratory findings, differential diagnoses of Intestinal tuberculosis and Crohn's disease were made.

**Figure 2.**
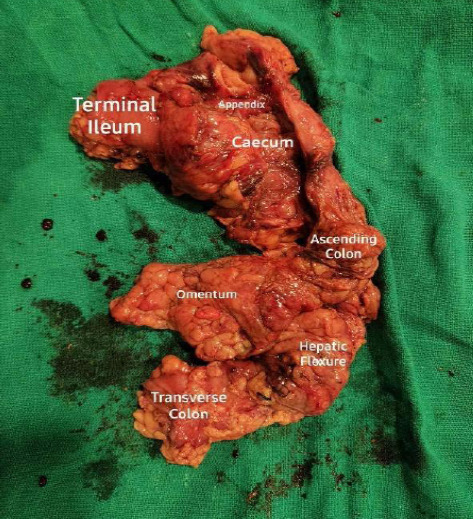
Resected Specimen-Right hemicolectomy showing stricture formation, areas of haemorrhage, and dense adhesion with surrounding mesentery.

We belong to the South Asian region where tuberculosis is more common than Crohn's disease.^6^ Therefore, Anti-tubercular therapy (ATT) was started empirically on clinical grounds, and the patient was advised to have a regular follow-up. The patient followed up weekly with an improvement of symptoms and also noticed weight gain. After the completion of two months of the intensive phase of ATT, the continuation phase of ATT was started. His fever, pain abdomen, weight loss, and bowel symptoms were improving for the initial 2 months but his gastrointestinal symptoms worsened over the next month. In the third month, however, he started having fever and pain in the right lower quadrant. Blood investigations were sent which came out to be normal and ultrasonography of the abdomen was done which showed a lump in the ileocecal region with multiple mesenteric lymph nodes. The patient had persistent severe abdominal pain for which NSAID (Non-steroidal anti-inflammatory drugs) was prescribed but the pain didn't subside. He was then referred for surgical consultation and a CT abdomen was performed which showed a circumferential wall thickening of the distal ileum and a lump in the ileocecal region with multiple mesenteric lymph nodes. With the informed consent of the patient, a right hemicolectomy was done (Figure 2).

Histopathological report of the specimen showed a granulomatous ulcerative ileal lesion with extensive peri-ileal adhesion, most likely Crohn's disease, and granulomatous lymphadenitis involving multiple lymph nodes most likely associated with Crohn's disease. The histopathology specimen slide was also consulted with multiple pathologists and was reported as multiple granulomas with Langhan's type multinucleated giant cells without caseous necrosis.

**Figure 3.**
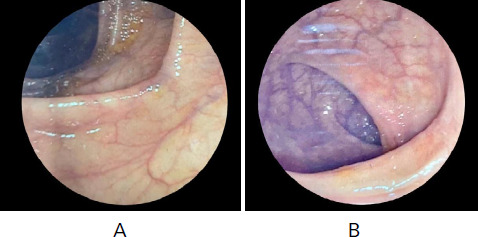
Colonoscopy showing A-normal-looking mucosa in the terminal ileum and B-anastomotic site.

After hemicolectomy and the histopathological reports, a diagnosis of Crohn's disease was made and ATT was stopped. The patient was treated symptomatically in the post-operative period and after 2 weeks of the operation, the patient was started on Azathioprine 50 mg orally once a day with close and regular follow-up. Two weeks later, the patient had improved appetite, and there was no fever, bowel movement was normal with normal well-formed stool. The dose of Azathioprine was increased to 75 mg once a day after 2 months. He showed significant improvement in his clinical condition. The patient was symptom-free and also had weight gain with a good appetite. After twelve months of treatment, a colonoscopy was done which showed a normal anastomotic site (Figure 3). Few erosions were seen in the terminal ileum and the rest of the colon was normal. Biopsy was done and the report showed focal active ileitis. Findings were mild and likely related to medications or anastomotic changes. Azathioprine was continued for the next 6 months and was tapered and stopped with a total duration of 20 months.

The last colonoscopy was done one year after the previous one normal-looking mucosa in the anastomotic site and normal mucosa in the terminal ileum. A biopsy was not done. The patient is in good general condition at present.

## DISCUSSION

CD is a chronic relapsing inflammatory bowel disease characterized by a transmural granulomatous inflammation that can affect any part of the gastrointestinal system.^8^ Yulan et al. found that relevant risk factors associated with CD are smoking, sanitation, appendectomy, certain medications like NSAIDs, nutrition (like fast food), stress, etc.^9^ However, there is no such exposure to risk factors in our patient as per his history. The presenting symptoms of patients are variable and diagnosis with signs and symptoms is very difficult. However, the patients often present with abdominal symptoms like abdominal pain, vomiting, and systemic symptoms like weight loss, low-grade fever, fatigue, etc. which were somewhat similar to our patient.^10^ It is crucial to distinguish between Intestinal TB and CD because the two conditions require very different therapies and immunosuppressive drugs for intestinal TB can have devastating effects.^5^

The various diagnostic tools for CD are laboratory investigations like fecal calprotectin, routine blood and stool examination, stool culture, C-reactive protein, tuberculosis screening; endoscopy and imaging; histopathology, etc.^11^ However, no pathognomonic markers for CD exist since all findings can also be seen in other diseases.^12^ Thus, while diagnosing CD, amebiasis, Behcet disease, Celiac disease, intestinal carcinoid, intestinal tuberculosis, mesenteric ischemia, ulcerative colitis, etc. should always be kept in mind.^1^

The task of an accurate diagnosis of CD in developing countries is further complicated because of the high prevalence of intestinal TB. In a given clinical situation, if the diagnosis of CD or intestinal TB can't be established, it is often presumed to be intestinal TB and the patient is treated accordingly.^13^ A response to ATT in such patients helps in the differential diagnosis but offers no scope of a definitive diagnosis of CD in nonresponsive patients. The lack of specific symptoms and a diagnostic test with high sensitivity and specificity for CD seems to be the reason for diagnostic failure. Such a situation is especially true for Nepal, which is a high-burden country for TB. About two-thirds of the total population is infected with TB and an estimated 20,000 new infectious cases are reported to occur each year. The estimated annual incidence rate for all types of tuberculous disease is 151 per 100 000 population, and the annual incidence of new smear-positive cases is 95 per 100 000 population which was similar to our case.^14^ Ibrahim et al. also mentioned that there is no such simple test for differentiating Intestinal TB and CD, as they share confusingly similar clinical, endoscopic, radiological, and pathological manifestations.^15^

Thus, our case was also treated with ATT considering the epidemiology of TB in our part of the world. Patients who do not respond to ATT but later respond to the treatment for CD should have a CD diagnosis taken into account. At 8-12 weeks after ATT, the clinical and colonoscopy response is evaluated. If the patient's symptoms improve, Intestinal TB is confirmed as the cause. ATT can be considered in regions where TB is endemic.^5^ The medical treatment modalities for CD can be done by oral mesalamine, immunomodulators, and biologics.^16^ Surgical treatment is often needed in fistulas, abscesses, perianal disease, etc. Anthony et al. found that surgical resections followed by prophylactic therapy were more effective in preventing recurrences. In our patient, a hemicolectomy was done.^17^ The patient is in good condition with a good appetite and weight gain.
